# Soybean Roots Grown under Heat Stress Show Global Changes in Their Transcriptional and Proteomic Profiles

**DOI:** 10.3389/fpls.2016.00517

**Published:** 2016-04-25

**Authors:** Oswaldo Valdés-López, Josef Batek, Nicolas Gomez-Hernandez, Cuong T. Nguyen, Mariel C. Isidra-Arellano, Ning Zhang, Trupti Joshi, Dong Xu, Kim K. Hixson, Karl K. Weitz, Joshua T. Aldrich, Ljiljana Paša-Tolić, Gary Stacey

**Affiliations:** ^1^Division of Plant Sciences and Biochemistry, National Center for Soybean Biotechnology, C.S. Bond Life Sciences Center, University of MissouriColumbia, MO, USA; ^2^Laboratorio de Genómica Funcional de Leguminosas, FES Iztacala Universidad Nacional Autónoma de MéxicoMéxico, Mexico; ^3^C.S. Bond Life Sciences Center, Informatics Institute, University of MissouriColumbia, MO, USA; ^4^Department of Computer Science, University of MissouriColumbia, MO, USA; ^5^Department of Molecular Microbiology and Immunology and Office of Research, School of Medicine, University of MissouriColumbia, MO, USA; ^6^Environmental Molecular Sciences Laboratory, Pacific Northwest National LaboratoryRichland, WA, USA

**Keywords:** soybean, root hairs, heat stress, gene module, transcriptomics, proteomics

## Abstract

Heat stress is likely to be a key factor in the negative impact of climate change on crop production. Heat stress significantly influences the functions of roots, which provide support, water, and nutrients to other plant organs. Likewise, roots play an important role in the establishment of symbiotic associations with different microorganisms. Despite the physiological relevance of roots, few studies have examined their response to heat stress. In this study, we performed genome-wide transcriptomic and proteomic analyses on isolated root hairs, which are a single, epidermal cell type, and compared their response to stripped roots. On average, we identified 1849 and 3091 genes differentially regulated in root hairs and stripped roots, respectively, in response to heat stress. Our gene regulatory module analysis identified 10 key modules that might control the majority of the transcriptional response to heat stress. We also conducted proteomic analysis on membrane fractions isolated from root hairs and compared these responses to stripped roots. These experiments identified a variety of proteins whose expression changed within 3 h of application of heat stress. Most of these proteins were predicted to play a significant role in thermo-tolerance, as well as in chromatin remodeling and post-transcriptional regulation. The data presented represent an in-depth analysis of the heat stress response of a single cell type in soybean.

## Introduction

Temperature is a critical factor that controls plant growth and development (Patel and Franklin, 2009). The Intergovernmental Panel on Climate Change (IPCC) has forecasted that global temperatures will increase between 2 and 5°C by the end of this century (http://www.ipcc.ch). In most regions, this global warming will negatively impact plant growth and development. As a consequence, the yields of a variety of important crops, such as corn, wheat, and soybean will be compromised. Thus, it is imperative to understand the physiological and molecular processes that plants use to cope with heat stress as a first step to breed for plants more tolerant to the negative effects of climate change.

Heat stress is considered one of the main factors that negatively affect crop production (Tubiello et al., 2007; Wheeler and von Braun, 2013). This is because high temperatures reduce plant growth, as well as the number of flowers and seeds per pod. At a biochemical level, high temperatures induce protein denaturation, increase membrane lipid fluidity, increase reactive oxygen species production, and inhibit function of the photosynthetic apparatus (Larkindale et al., 2005; Hasanuzzaman et al., 2013; Qu et al., 2013). Plants have developed a variety of adaptations that allow them to cope with heat stress. Some of these responses include changes in leaf orientation, modification of membrane lipid composition, activation of anti-oxidative mechanisms, accumulation of osmolites, and early maturation (Hasanuzzaman et al., 2013). These responses are finely regulated at transcriptional, post-transcriptional, and post-translational levels by different transcription factors (TFs), small RNAs, and protein kinases, respectively (Chen et al., 2012; Guan et al., 2013; Sullivan et al., 2014).

Soybean is a chief source of protein for human consumption and is grown on about 6% of the world's arable lands (Hartman et al., 2011). Soybean production in the United States significantly increased over the last 10 years with a concomitant increase in the value of the crop. However, as a clear example of the impact of abiotic stress, US soybean production was reduced ~7% during the severe drought/heat period in 2012 (http://www.ers.usda.gov/topics/in-the-news/us-drought-2012-farm-and-food-impacts.aspx#crop). Useful models to predict the impacts of changing climate on plant productivity will require accurate, quantitative data that predict impacts across broad levels and spatial scales. The combination of systems biology and different “omics” approaches (i.e., transcriptomic, proteomic, and metabolomics) offers an attractive option to study the impact of global climate change on plant growth and development. Moreover, the inclusion of single cell models can significantly increase the accuracy and resolution of this approach, especially by avoiding signal dilution.

Most studies of plant responses to heat stress have focused mainly on above ground organs. Here, we focus our evaluation of heat stress on root cells and tissues. Roots provide support, water and nutrients to other plant organs (Khan et al., 2011). Indeed, soil temperature can influence root growth, cell elongation, root length and extension, initiation of new lateral roots and root hairs, and root branching (Pregitzer et al., 2000). These effects are likely manifestations of the variety of physiological effects brought about by temperature on plant roots; including changes in root respiration, nutrient uptake, as well as physicochemical effects on the soil environment (e.g., changes in nitrogen mineralization). Ambient temperature changes on above ground plant organs (e.g., effects on photosynthetic rates) also affect below ground growth and physiology. Despite the physiological relevance of roots, few studies have examined the response of these plant organs to heat stress.

In this study, we analyzed the transcriptional and proteomic responses of soybean roots to heat stress. In order to better understand the root responses to this abiotic stress, we performed genome-wide transcriptomic and proteomic analyses on root hairs, which are a single epidermal cell type. Our transcriptional analysis identified 1849 genes differentially regulated in root hairs in response to heat stress. These data were used to predict key regulatory modules controlling the heat stress response. We also conducted proteomic analysis on membrane fractions isolated from stripped roots and root hairs. These experiments identified a variety of proteins whose expression changed within 3 h of application of heat stress. Most of these proteins were predicted to play a significant role in thermo-tolerance, as well as chromatin remodeling and post-transcriptional regulation. The data presented represent an in-depth analysis of the heat stress response of a single cell type in soybean.

## Materials and methods

### Plant material and treatments

Soybean seeds [*Glycine max* L. (Merrill) cv. Williams 82] were surface sterilized and sown on agar plates containing 1X B&D (Broughton and Dilworth, 1971) nutrients. Plates containing seeds were incubated for 3 days under dark conditions at 25°C in a growth chamber. Three day-old seedlings were further incubated for various time points (0, 3, 6, 12, and 24 h) at 25°C (control) or 40°C (heat stress). After a specific incubation time, the whole roots were detached from the shoots and immediately frozen in liquid nitrogen. These roots were used to isolate root hairs and corresponding stripped roots (i.e., roots with root hairs removed) according to the methods described in Brechenmacher et al. (2009). Once the root hairs (RHs) were removed from the whole root, both frozen stripped roots (STRs) and RHs were stored at –80°C until use. Two biological replicates per time point were collected. In each biological replicate, 50 plates (each plate contained 20 seeds, five plates for each time, and temperature condition, in total 1000 seedlings were used in each biological replicate) were included.

### Protein and RNA extraction

Proteins and total RNA were extracted from 1 g of RHs or STRs using Trizol reagent supplemented with protease inhibitors according to the manufacturer's instructions. Total RNA was subsequently purified using a chloroform extraction. Total RNA concentration and integrity were analyzed using a Nanodrop (Thermo Scientific, Whilmington, DE) analyzer and a Bioanalyzer (Agilent, Santa Clara, CA), respectively. A Coomassie Plus (Thermo Scientific, Grand Island, NY) protein assay was used to quantify the total protein concentration and about 200 μg of protein per sample were obtained.

### Microsomal fraction

The microsomal fraction was purified from RH or STR extracts according to Brechenmacher et al. (2009). Briefly, homogenized RH preparations were sonicated in 0.1 M Tris–HCl, pH 8, 10 mM EDTA, 0.4% β-mercaptoethanol, and 250 mM sucrose. Cell debris was allowed to settle and organelles removed from the suspension by centrifugation at 20,000 g for 30 min at 4°C. The microsomal fraction was obtained by centrifugation at 100,000 g for 1 h at 4°C. The pellets were solubilized in 0.1 M Tris–HCl, pH 8.5, 8 M urea, and 2% dodecyl-β-maltoside. Proteins were precipitated using 25% trichloroacetic acid (TCA), washed in acetone, and resolubilized with 8 M urea and Tris–HCl, pH 8.5. Finally, the supernatants were passed through 5.0 and 0.45-μM polyvinylidene difloride membrane filters (Millipore, Billerica, MA). Proteins were quantified using the bicinchoninic acid protein assay kit (Thermo Scientific, Grand Island, NY). The same procedure was used to obtain microsomal fractions from homogenized STR preparations, except that the roots were ground using a mortar and pestle to extract the protein.

### Protein sample preparation and LC-MS/MS analysis

The extracted microsomal proteins for three independent sample for both RHs and STRs were dried with a speed vac, followed by solubilization and denaturation in 150 μL of 7 M urea, 2 M thiourea, 4% 3-[(3-cholamidopropyl)dimethylammonio]1-propanesulfonate (CHAPS) and 5 mM Tris(2-carboxyethyl)phosphine (TCEP) in 50 mM ammonium bicarbonate, pH 8. These preparations were vortexed, sonicated and then heated at 60°C for 30 min. Protein concentrations were again verified using the Coomassie Plus Protein Assay with a bovine serum albumin standard. The denatured samples were diluted 10-fold with 50 mM ammonium bicarbonate. CaCl_2_ was added to a concentration of 2 mM and trypsin was added at a trypsin:sample ratio of 1:50 (w/w). The samples were digested overnight at 37°C and were alkylated with chloroacetamide at a concentration of 5 mM in the dark for 2 h at room temperature (RT). The peptides were desalted using SCX SPE resin (SUPELCO Supelclean, 100 mg) using first a 10 mM ammonium formate, pH 3.0, 25% acetonitrile solution to wash the peptides followed by 80:15:5 methanol:water:ammonium hydroxide to elute the peptides. The SCX SPE resin removed the detergents but the ammonium salts still needed to be removed before iTRAQ labeling. For this latter purpose, the samples were loaded onto a C-18 SPE column (SUPELCO Discovery, 50 mg) followed by a wash using 0.1% TFA in nanopure water and then subsequently 80% acetonitrile/0.1% TFA in water to elute the peptides. Peptides were quantified using a BCA assay with a bovine serum albumin standard.

Peptides were labeled with 8-plex iTRAQ reagents as described below (AB Sciex, Foster City, CA). Thirty micrograms of each sample was placed in a new tube and dried in a speedvac. Thirteen micrograms of dissolution buffer (provided in the iTRAQ kit) was added to each sample and vortexed into solution followed by brief centrifugation to concentrate sample at the bottom of the tube. Each iTRAQ reagent (10 μL) was diluted with isopropanol (35 μL) and then added to each sample. The reaction was carried out for 2 h at RT. Fifty millimolars of ammonium bicarbonate (200 μL) was added to quench each reaction tube. After 1 h, the contents from all iTRAQ reactions were added to one tube and the sample was vortexed, followed by drying in a speed vac.

The labeled peptides were separated using an off-line high pH (pH 10) reversed-phase (RP) XBridge C18 column (Waters, Milford MA; 250 × 4.6 mm column containing 5 μm particles and a 4.6 × 20 mm guard column) using an Agilent 1200 HPLC System (Agilent Technologies, Santa Clara CA). The sample loaded onto the C18 column was washed for 15 min with Solvent A (10 mM ammonium formate, adjusted to pH 10 with ammonium hydroxide). The LC gradient used a linear increase of Solvent B (10 mM ammonium formate, pH 10, 90% acetonitrile) to 5% over 10 min, then a linear increase to 45% Solvent B over 65 min, and then a linear increase to 100% Solvent B over 15 min. This level of Solvent B was held at 100% for 10 min and subsequently dropped to 0% Solvent B, holding the column at 100% Solvent A for 20 min. The flow rate was 0.5 mL/min. A total of 48 fractions (1.98 mL each) were collected evenly over the gradient between 15 and 110 min into a deep (2 mL/well) 96 well plate throughout the LC gradient. The plate fractions were concentrated using a speed vac. The high pH RP fractions were then combined into 12 fractions using the concatenation strategy reported in a previous study (Wang et al., 2011) which were further dried down and resuspended in nanopure water at a concentration of 0.075 μg/μL. Fractions were stored at −20°C until time for LC-MS/MS analysis.

Peptide mixtures were analyzed on a high-resolution, reversed-phase constant flow nano capillary LC system coupled to an LTQ Orbitrap Velos mass spectrometer (Thermo Scientific, San Jose CA). The automated LC system was custom built using two Agilent 1200 nanoflow pumps and one Agilent 1200 Capillary pump (Agilent Technologies, Santa Clara CA), and a PAL® autosampler (LEAP Technologies, Carrboro, NC). Full automation was made possible by custom software allowing for parallel event coordination. Therefore, 100% of the MS duty cycle was used by way of two trapping and two analytical capillary columns. Capillary reversed-phase columns were prepared in-house by slurry packing 3-μm Jupiter C18 (Phenomenex, Torrence, CA) into 35 cm × 360 μm o.d. × 75 μm i.d. fused silica (Polymicro Technologies Inc., Phoenix AZ). Trapping columns were prepared similarly, but using 3.6 μm Aeris Widepore XB-C18 resin packed into a 4 cm length of 150 μm i.d. fused silica. Electrospray emitters were custom made using 150 μm o.d. × 20 μm i.d. chemically etched fused silica (Kelly et al., 2006). Mobile phases consisted of 0.1% formic acid in water (A) and 0.1% formic acid acetonitrile (B) operated at 300 nL/min with a gradient profile as follows (min:%B); 0:5, 2:8, 20:12, 75:35, 97:60, 100:85. Sample injections (5 μL) were trapped and washed on the trapping columns at 1.5 μL/min for 20 min before alignment with the analytical columns.

The LTQ Orbitrap Velos mass spectrometer was operated with a heated capillary temperature and spray voltage of 350°C and 2.2 kV, respectively. Full MS spectra were recorded at a resolution of 100 K (for ions at *m/z* 400) over the range of *m/z* 400–2000 with an automated gain control (AGC) value of 1e6. MS/MS was performed in the data-dependent mode with an AGC target value of 3e4. The 10 most abundant parent ions, excluding singly charged ions, were selected for MS/MS using high-energy collisional dissociation (HCD) with a normalized collision energy setting of 40%. A dynamic exclusion time of 45 s was used.

### Identification of differentially expressed proteins

MS/MS spectra were first converted to peak lists using DeconMSn (version 2.2.2.2, http://omics.pnl.gov/software/DeconMSn.php) (v1) with default parameters. Sequence determination was provided by SEQUEST v27 in conjunction with the soybean genome annotation (Gmax v10.3;Wm82.a2.v1). Both full and partially digested tryptic peptides were considered with two missed cleavages allowed. The mass tolerance for precursor ions was 50 ppm and fragmentation tolerance for HCD (higher energy collisional dissociation) was 0.05 Da. All peptides were identified with < 1% False Discovery Rate by using an MS-Generating Function Score (MS-GF) < 1e-10 and a decoy database searching strategy (Kim et al., 2008, 2010; Granholm et al., 2014). Modifications were searched looking for static alkylation on cysteine and 8 plex iTRAQ modifications on the N-terminus and lysine residues. Other modifications included in the search were dynamic oxidation on methionine. Relative abundances of peptides were determined using iTRAQ reporter ion intensity ratios from each MS/MS spectrum. Individual peptide intensity values were determined by dividing the base peak intensity by the fraction associated with each reporter ion. Multiple scans of the same peptide were consolidated into a single peptide value by summation. Log2 transformed peptide abundances were then normalized according to the mean of the two pooled references (added to two channels of each iTRAQ 8-plex experiment) so that samples from different iTRAQ experiments could be compared. The peptide abundances were further normalized (to remove iTRAQ channel bias) using the central tendency normalization algorithm (which normalizes each proteome dataset to the global population median) available in Inferno (http://code.google.com/p/inferno4proteomics/). The Rrollup function in Inferno was used to roll up peptide values to a protein value. The Rrollup function works by taking log2-transformed data and identifying the peptide which has the most presence and greatest abundance across all samples used for comparison. All peptides were scaled to the most present and abundant peptide and the final protein abundance value used represents the median of the scaled peptide abundances. ANOVA significance testing was performed on each sample time point, determining significance via *p*-value between samples subjected to either 25 or 40°C. *P*-values were further corrected for multiple-testing error using Benjamini–Hochberg *p*-value correction. Fold changes are displayed as log2 fold change of protein values obtained at 40°C/protein values obtained at 25°C. The Figure S5 shows a detailed workflow about the design of this experiment.

### Preparation of RNA-seq library

Total RNA was isolated from 1 g of control- or heat-stressed RHs or STRs as described above. Non-strand-specific mRNA-seq libraries were generated from 4 μg of total RNA from each tissue and prepared using the TruSeq RNA sample Prep Kit (Illumina) according to the manufacturer's instructions.

### High-throughput transcriptomics sequencing

Forty non-strand-specific RNA-seq libraries 2 root types (RH and STR) × 2 treatments (Control or Heat stress) × 5 treatments × 2 replicates] were multiplexed and sequenced for 51 cycles using an Illumina HiSeq 2000 (Illumina, San Diego, CA) according to the manufacturer's instructions. Image analysis and base calling were performed using the Illumina pipeline (http://www.illumina.com).

### Mapping and processing of RNA-Seq reads

Following base calling, quality filtering was performed on the RNA-Seq reads generated with the Illumina HiSeq 2000 using an in-house custom Perl script including removal of reads with “N” base and trimming of the 3′-end of the read for below threshold quality. Additional filtering for removal of bad-quality bases (any base with quality score values lower than 20 percentile was considered as a base with bad quality) and read length size (< 50 bp) was performed using the FASTA/Q Trimmer command of the FASTX-toolkit available in FastQC software package (http://www.bioinformatics.babraham.ac.uk/projects/fastqc/). RNA-seq reads with good quality were aligned to the soybean reference genome (Gmax v10.3;Wm82.a2.v1; Schmutz et al., 2010) using Tophat (version 1.4.1; Trapnell et al., 2009). The genome indexes for Tophat were built using bowtie-build command of bowtie (version 0.12.7) with the reference genome file as the input. Tophat was then run with the default parameters to map the trimmed- and filtered-reads for each library to the reference genome. Tophat was supplied with the reference GTF file using the –G option and replicates of each condition/sample were mapped independently to improve alignment sensitivity and accuracy for further analysis. For analysis of protein-coding genes, only uniquely mapping reads were used. The gene expression abundance was calculated in RPKM using Cufflinks software (Trapnell et al., 2012).

### Identification of differentially expressed genes

Low-count reads with a total sum fewer than 10 were removed prior to data analysis (Auer and Doerge, 2011). A Poisson linear mixed-effects model (Blekhman et al., 2010) was applied to the raw read counts separately for each gene using the software R/lme4 package (2.10.0 version) with the library size as the offset value to make the comparison across different samples comparable. Each generalized Poisson linear mixed model includes the cell type effect, treatment effect, and the random biological replicate effect, as well as random plate effect accounting for the correlation between observations that share the same plate. The likelihood ratio tests were then conducted to identify differentially expressed genes between the treatment and control groups for each of the cell types (i.e., RH_heat_ vs. RH_control_). *P*-values for the likelihood ratio tests were obtained, and an adjusted-*P*-value (Storey and Tibshirani, 2003) was then computed to produce lists of differentially expressed genes with an estimated FDR of 1%. Among these significantly differentially expressed genes, genes with a fold change above two were further considered.

### Gene regulatory networks analysis

A gene regulatory module analysis was performed using the method described by Zhu et al. (2012). Briefly, based on the differentially regulated genes between the treatment and control groups for each exposition time (i.e., RH_heat_ vs. RH_control_), the expression levels of transcription factors (TF) were clustered into two or three categories (1: highly expressed; 0: normally expressed; –1: lowly expressed) using the K-means clustering algorithm, where the number of categories equals the number of types (>3, < –3, or in between) of expression. A specific set of TFs was assumed to regulate the expression of genes in a module through a path in the binary decision tree composed of TFs as internal nodes and condition subgroups as leaf nodes. A regulatory path from the root node to the leaf node was interpreted as a series of binary queries on the expression level (up-regulated or not, or down-regulated or not) of internal nodes (i.e., TFs) under treatment conditions leading to the observed expression levels of the genes in the leaf node under the same treatment conditions. Therefore, the regulatory decision tree represents the combinatorial logic by which the TFs regulate the expression of the genes in the module under different treatment conditions. In order to construct the gene regulatory module, the differentially expressed genes were clustered using the K-means algorithm, aiming to assign genes exhibiting similar expression patterns across all the treatment conditions into the same cluster. Once the genes were clustered, the modules were constructed in an iterative two-step manner, including: (1) constructing a binary tree consisting of several TFs that can best interpret the expression of the genes in a cluster, and (2) re-assigning into clusters those genes whose regulatory tree can explain their expression pattern best. The two steps were alternated until the likelihood of the gene expression data was maximized. After a gene regulatory tree was constructed for every gene cluster, a gene re-assignment procedure was used to assign each gene to a cluster whose regulatory tree best explained its expression values over all the treatment conditions.

### Gene functional classification

The biological relevance of the differential regulated genes and proteins was assessed by a gene function enrichment analysis using the method Singular Enrichment Analysis (SEA) available in the web-based tool AgriGo (Du et al., 2010; http://bioinfo.cau.edu.cn/agriGO/analysis.php). Briefly, Gene Ontology (GO) terms enriched in each individual set of genes and proteins were compared to the Wm82.a.V.2.1 gene reference background. *P*-values for the GO terms were obtained through Fisher's exact test, and a *q*-value was computed to produce lists of significant GO terms with an estimated FDR of 5%. Among these significantly enriched GO terms, terms with *q* > 0.05 were further considered. Additionally, MAPMAN gene functional classification was used (Thimm et al., 2004; Usadel et al., 2009). For MAPMAN analysis an in-house custom soybean mapping file, which allows a survey of all the functional categories included in the software MAPMAN, was used.

### RNA extraction and qRT-PCR

Total RNA was isolated from stressed or control RHs and STR using Trizol Reagent (Invitrogen), according to manufacturers' specifications. Genomic DNA (gDNA) was removed from purified RNA by using TURBO DNAse (Ambion) according to manufacturer's instructions. Two micrograms of gDNA-free RNA were used to synthetize cDNA as described in (Libault et al., 2010).

qRT-PCR was performed as described in Libault et al. (2008) using the housekeeping gene *cons6* to normalize the expression levels of the analyzed genes (Libault et al., 2008). Primer design was performed as described in Libault et al. (2010). Expression levels of each candidate gene were calculated according to E = ${\text{P}}_{\text{eff}}^{\left(-\Delta \text{Ct}\right)}$, where P_eff_ is the primer efficiency calculated using LinRegPCR (Ramakers et al., 2003). Fold changes were calculated between the ratios of the expression levels of heat-treated (40°C) and control (25°C) samples, and expression levels were calculated for three different time points (3, 12, and 24 h) for two biological replicates.

## Results

### RNA-seq analysis

Most studies of the transcriptional responses to heat stress used entire organs (e.g., leaves) (Barah et al., 2013; Johnson et al., 2014). Thus, the values obtained from these studies represent an average of the response of all the different cell types in the tissue analyzed. In order to reduce the “tissue dilution” effects inherent to such studies, we conducted an RNA-seq analysis using a single-type of soybean cell, the root hair.

A total of 40 cDNA libraries were derived from control RHs (RH_C) and RHs exposed to 40°C (RH_H), as well as from control STRs (STR_C) and STRs exposed to 40°C (STR_H). These libraries were sequenced using the Illumina HiSeq2000 platform. After filtering low quality reads, a total of 1,053,532,578 reads (50 bp in size) were aligned to the soybean genome reference sequence (Gmax v10.3;Wm82.a2.v1; Schmutz et al., 2010; Table S1) using Bowtie and Tophat software (Trapnell et al., 2009). Of these, 997,923,404 reads were uniquely mapped to the soybean genome and were used for further analysis. Of the 56,044 predicted protein-coding genes in the soybean genome (https://phytozome.jgi.doe.gov/pz/portal.html), 46,366 genes were deemed to be expressed in this study based on the occurrence of at least one read in both biological replicates (Table S1). The RNAseq gene expression, proteomic expression, and differential expression datasets are all available for browsing in the Soybean Knowledge Base (SoyKB; http://www.soykb.org/; Joshi et al., 2012, 2014).

### Transcriptional responses to heat stress at single cell resolution

**Figure 1:**
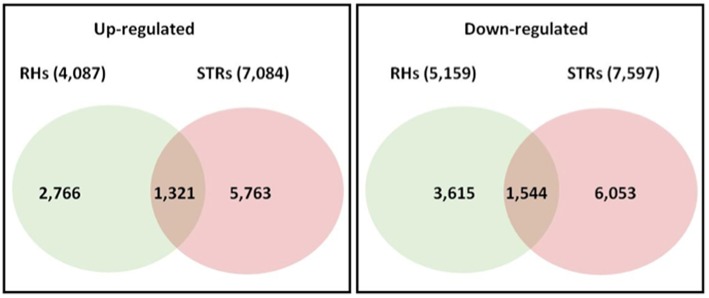
**Number of overlapping and non-overlapping heat-responsive genes among soybean root hairs (RHs) and stripped roots (STRs)**. Differentially regulated genes in each cell type were identified by linear mixed models at FDR < 0.01, with additional cutoff of two-fold in pairwise comparison (heat-stressed vs. control). Over- and non-over-lapping genes were identified after a pairwise comparison between treatments. Numbers in parenthesis indicate all the regulated genes across the four exposure time points.

In order to identify genes that were differentially regulated in response to heat stress, the RNA-seq data were analyzed using a generalized Poisson linear mixed-effects model with an additional cutoff of 2-fold in pairwise comparisons (e.g., RH_H/RH_C). On average, 1849 regulated genes were identified in RHs, with a maximum of 3126 (3 h treatment: 1447 up-regulated and 1679 down-regulated). In contrast, on average, 3091 regulated genes were identified in STRs, with a maximum of 4484 (6 h treatment: 2210 up-regulated and 2274 down-regulated). Across all four-exposure time points (3, 6, 12, and 24 h) to the heat stress, a total of 9246 and 14,681 differentially regulated genes were identified in RHs and STRs, respectively (Figure S1). A comparison across the four-treatments revealed 4087 genes that were up-regulated in RHs, whereas 7084 were up-regulated in STRs (Figure S1). Subsequently, a comparison between the differentially regulated genes in both RH and STR samples was performed to identify genes commonly or uniquely regulated in these tissues. This analysis revealed that 2865 genes (1321 up-regulated and 1544 down-regulated) were commonly regulated in both RHs and STRs (Figure 1 and Table S1). In contrast, 6381 (2766 up-regulated and 3615 down-regulated) and 11,816 (5763 up-regulated and 6053 down-regulated) were uniquely regulated in RHs and STR, respectively (Figure 1). These numbers attest to the strong impact that heat treatment has on transcription.

To confirm these RNAseq results, the expression of 15 randomly selected genes was analyzed via qRT-PCR (Figure S2). The pattern of expression obtained by qRT-PCR showed the same trend observed by RNAseq in response to heat stress. We found some differences in the fold-change measured by the two methods, which we explain by the relative sensitivity of each method, as well as technical aspects (e.g., efficiency and specificity of qRT-PCR primers).

We also compared the transcriptional response in RH and STR across the four treatment time points (3, 6, 12, and 24 h). This analysis revealed that only 14% (645 genes: 330 up-regulated and 315 down-regulated) of the differentially regulated genes in RHs were regulated across all four-treatment time points. In the STR samples, only 13% (1026 genes: 530 up-regulated and 496 down-regulated) of the differentially regulated genes were regulated at all four treatment time points (Figure S1). Collectively, these data indicate that each root-tissue type responded differently to the heat stress.

Transcriptional responses to heat stress might be controlled by 10 different regulatory modules in RHs.

Recently, we developed a new algorithm that can predict gene regulatory modules from either DNA microarray or RNA-seq transcription data (Zhu et al., 2012, 2013). This allows prediction of: (1) transcription factors (TFs) that control a specific regulatory module; and (2) genes that participate in a specific regulatory module (Zhu et al., 2012, 2013). We used this algorithm to analyze the differentially regulated genes that responded across all four-treatment time points (i.e., 645 commonly regulated genes). This analysis predicted 10 different regulatory networks (Figure S3). These modules are regulated in a combinatorial manner by five TFs: Heat Stress Factor (HSF; Glyma.03G157300), AP2/EREBP (Glyma.13G152000), MAD-box (Glyma.07G181600), and two WRKYs TFs (Glyma.17G097900 and Glyma.19G020600). With the exception of the HSF, the expression of the TFs that control the 10 gene regulatory modules was down-regulated across the four treatments. Furthermore, we found that five of the 10 regulatory networks contain down-regulated genes, whereas the other five networks have either highly- (fold-change ≥3) or mildly (fold-change ≤ 2) up-regulated genes. Interestingly, the TF WRKY encoded by Glyma.17G097900, regulates eight of the 10 modules, which indicates that this TF may be a master regulator of the heat stress response in soybean RHs.

**Figure 2:**
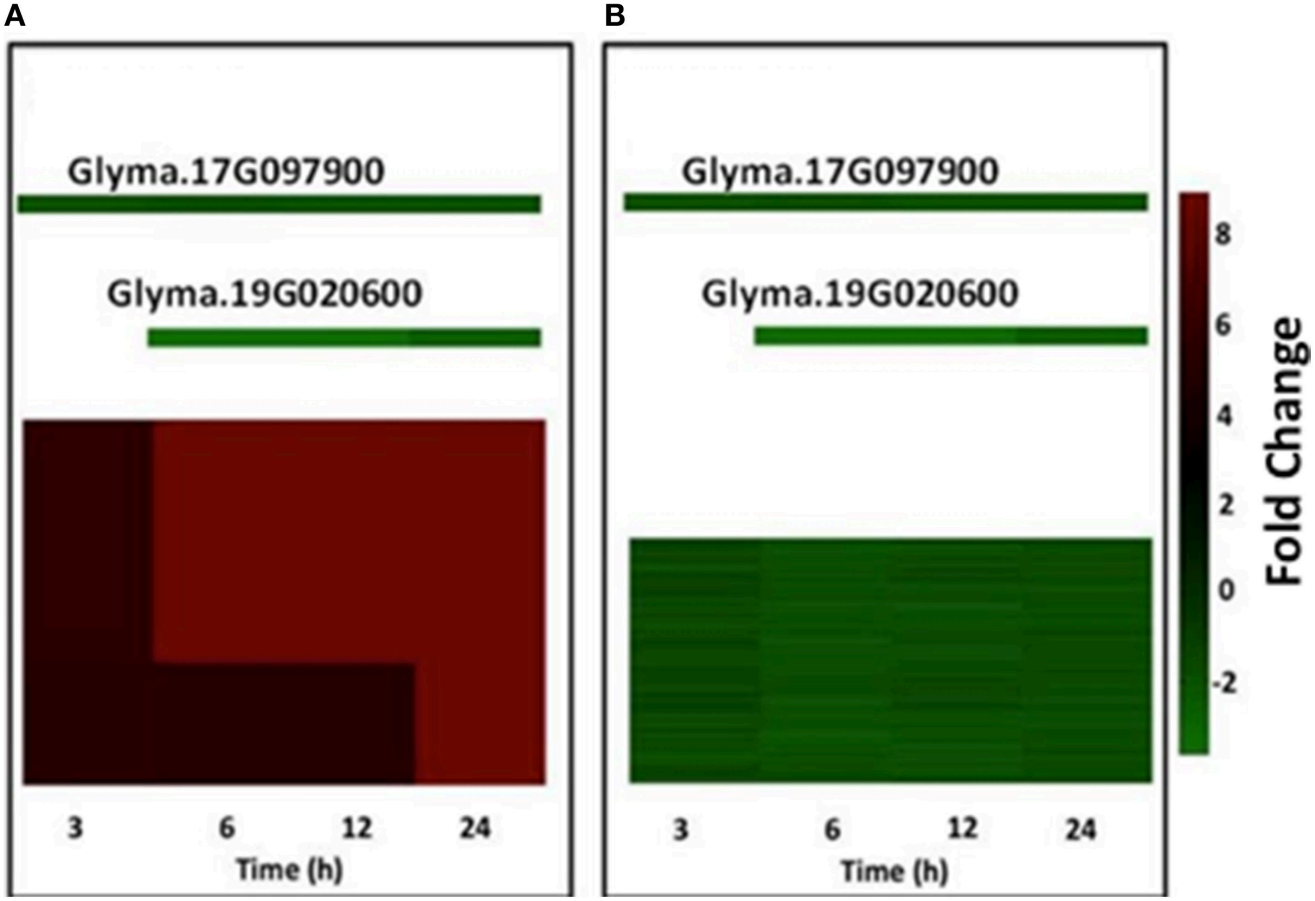
**Gene regulatory modules controlling the transcriptional responses of soybean root hairs to heat stress**. Panels **(A,B)** show two significant modules controlled by two different TFs. Individual genes are represented by small squares. Transcript abundance values were false color-coded by using a scale of −2 to ±8. The intensity of green and red colors indicates the degree of expression of the corresponding genes. Data are the average of two biological replicates.

By way of an example, we describe in detail the regulatory modules 7 and 9 in Figure 2. Module 7 contains two signal-transduction related genes (Glyma.14G100800: Receptor kinase; Glyma.15G048500: MAPKKK) whose expression was up-regulated across the four treatments (Figure 2A). These genes are predicted to be under the control of two WRKY TFs (Glyma.17G097900 and Glyma.19G020600) whose expression was down-regulated across the four treatments. Module 9 contains 84 down-regulated genes controlled by the same two WRKY TFs that control module 7. Some of the genes belonging to this network are likely involved in cell wall degradation, flavonoid biosynthesis and transcriptional regulation by different TFs, like MYB and C2H2 (Figure 2B). Collectively, the gene network analysis suggests that many heat-stress induced genes might be significantly regulated by only five different TFs. Furthermore, four (i.e., Glyma.17G097900, Glyma.19G020600, Glyma.13G152000, and Glyma07G181600) of these five TFs likely act as repressors of gene expression. However, further investigation is needed to validate these predictions.

### Heat stress induces changes in the root hair proteome

**Figure 3:**
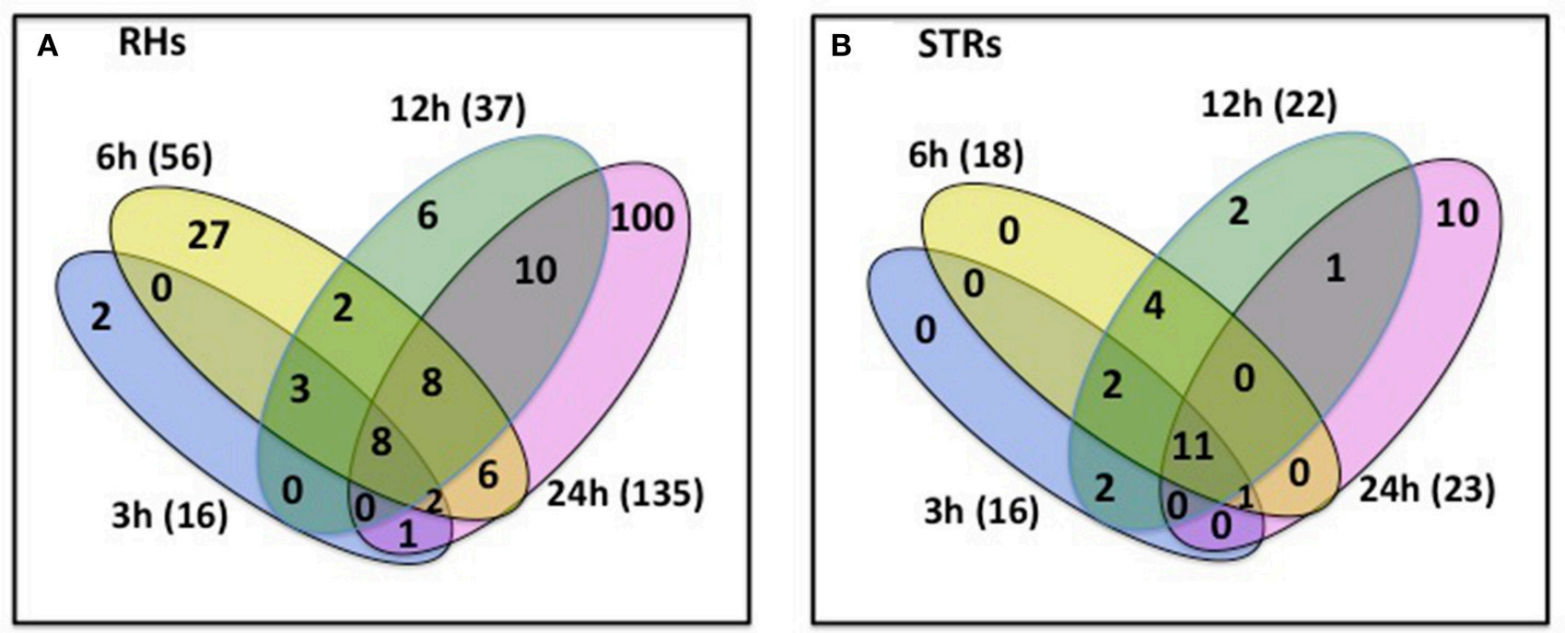
**Number of overlapping and non-overlapping heat-stress responsive proteins detected at four exposure times in soybean RHs (A) or STRs (B)**. Differentially regulated proteins in each cell type were detected by LC/MS/MS at FDR < 0.05; Fold Change >2. Over- and non-overlapping proteins were identified after a pairwise analysis. Gene identification for each gene bellowing to each category is provided in the Table S3.

**Figure 4:**
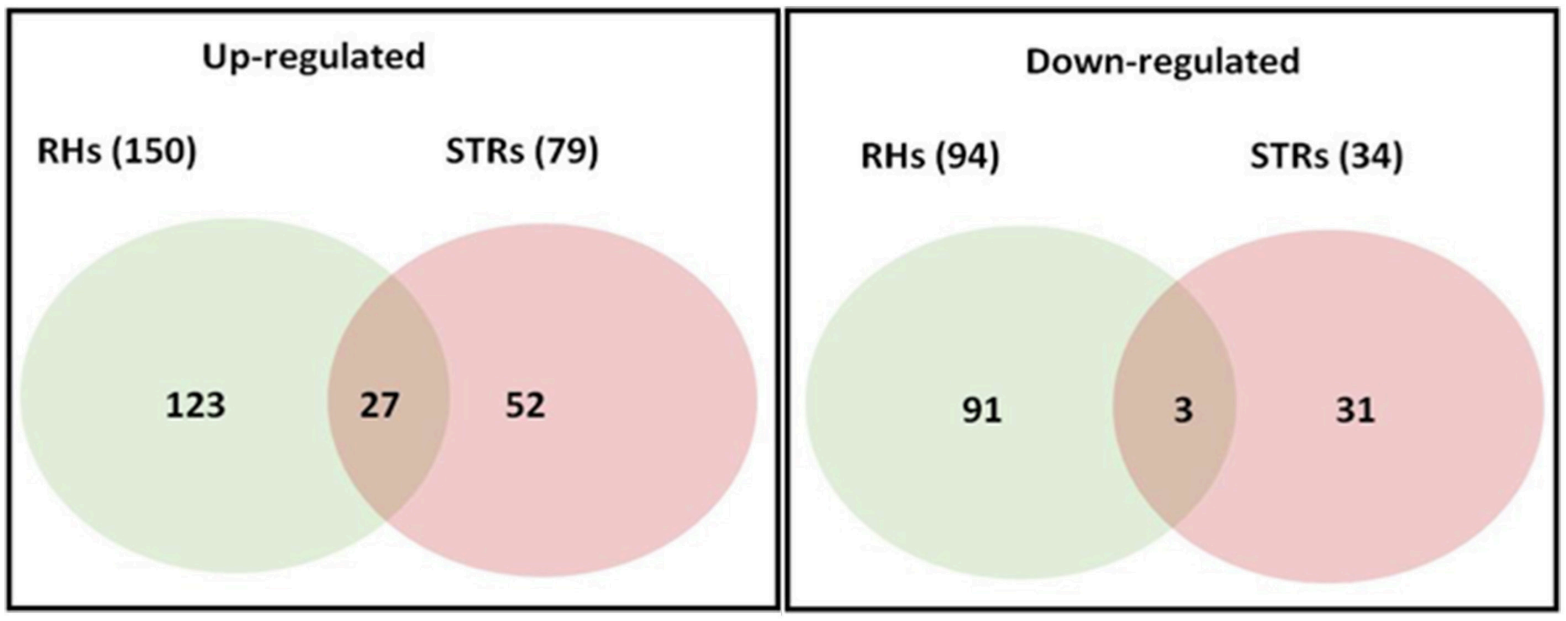
**Number of overlapping and non-overlapping heat-stress responsive proteins among soybean RHs and STRs**. Differentially regulated proteins in each cell type were detected by LC/MS/MS at FDR < 0.05; Fold Change >2. Over- and non-overlapping proteins were identified after a pairwise analysis. Gene identification for each gene bellowing to each category is provided in the Table S4.

Changes in transcriptional activity do not always reflect changes in expression of the encoded proteins (Stevens and Brown, 2013; Ponnala et al., 2014). Therefore, we also undertook an analysis of the RHs and STRs proteome in response to heat stress. We specifically focused on the microsomal (membrane) fraction in order to reduce the complexity of the protein profile and to specifically determine how membrane function (e.g., transporter expression) was affected by heat stress. Proteins were isolated from the same RHs and STRs used for the RNA-seq transcriptome analysis. LC/MS-MS analysis of these samples identified 244 and 79 differentially expressed proteins in RHs and STRs, respectively (Figure 3). To identify commonly or uniquely regulated proteins, we performed a comparative analysis among the proteins detected in both tissue types. Our analysis revealed that only 30 proteins (27 up-regulated and three down-regulated) were commonly expressed in response to heat in both RHs and STRs (Figure 4). In contrast, 214 proteins (123 up-regulated and 91 down-regulated) were expressed only in RHs whereas 83 (52 up-regulated and 31 down-regulated) were exclusively expressed in STRs (Figure 4). We observed that 3 h of heat treatment was sufficient to trigger significant changes at protein levels in response to heat stress. Additionally, we detected more differentially expressed proteins in RHs than in STRs. For instance, 135 differentially regulated proteins (73 induced and 62 repressed) were detected in RHs after 24 h of treatment, whereas 43 (23 induced and 20 repressed) were detected in STRs. This is likely due to a reduction in the effects of tissue dilution (that averages the signal over many cell types) in the STR samples, relative to the RHs. The proteomic expression and differential expression data are available for browsing in the Soybean Knowledge Base (SoyKB; http://www.soykb.org/; Joshi et al., 2012, 2014).

### Heat-stress related proteins play some role in the chromatin remodeling and post-transcriptional regulation in RHs

**Figure 5:**
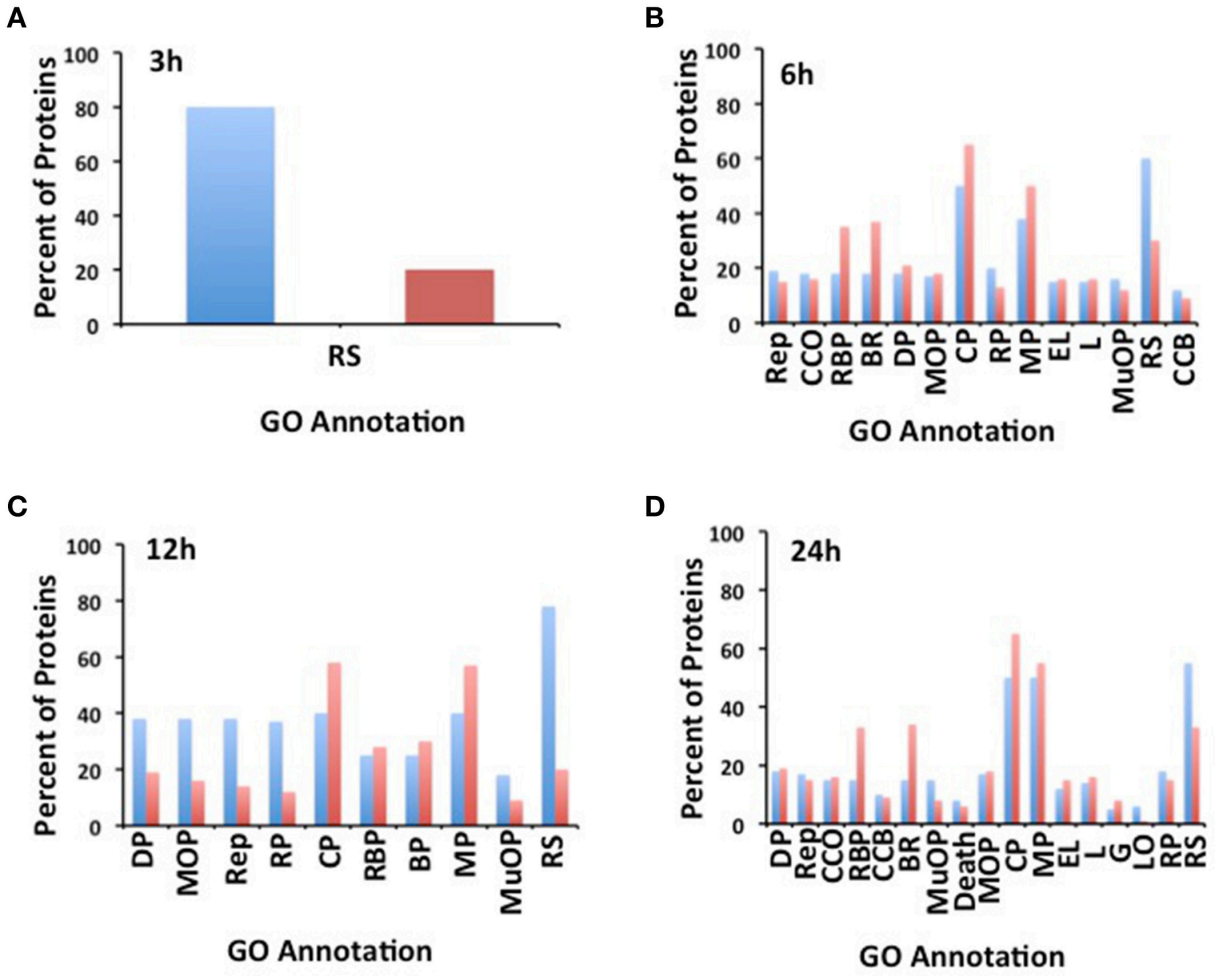
**Gene Ontology (GO) enriched terms of the differentially regulated genes identified in soybean root hairs (A, 3 h; B, 6 h; C, 12 h; and D, 24 h)**. The GO annotation is: RS: Response to Stimulus; Rep: Reproduction; CCO: Cellular Component Organization; RBP: Regulation of Biological Process; BR: Biological Regulation; DP: Development Process; MOP: Multicelluar Organismal Process; CP: Cellular Process; RP: Reproductive Process; MP: Metabolic Process; EL: Establishment of Localization; L: Localization; MuOP: Multi-organism Process; CCB: Cellular Component Biogenesis; G: Growth; LO: Locomotion.

A functional enrichment analysis, as well as a functional classification by using MAPMAN software, was performed on the regulated proteins identified in each heat treatment (Figure 5 and Figure S4). This analysis revealed that proteins known to respond to environmental stimuli that includes response to light, temperature, and heat stress, were those most enriched among the proteins responding to the heat treatment. This category was followed by proteins involved in cellular and metabolic processes, for instance in cell wall formation, amino acids, and lipid biosynthesis as well as in the elimination of reactive oxygen species (Figure 5). Interestingly, 84% of the identified proteins responding after 3 h of treatment were related to the heat stress response. Although, proteins with a potential role in adaptation to heat stress were identified at the other treatment time points, proteins with a potential role in chromatin remodeling, post-transcriptional and post-translational regulation were also identified in these time points (Figure 5).

**Figure 6:**
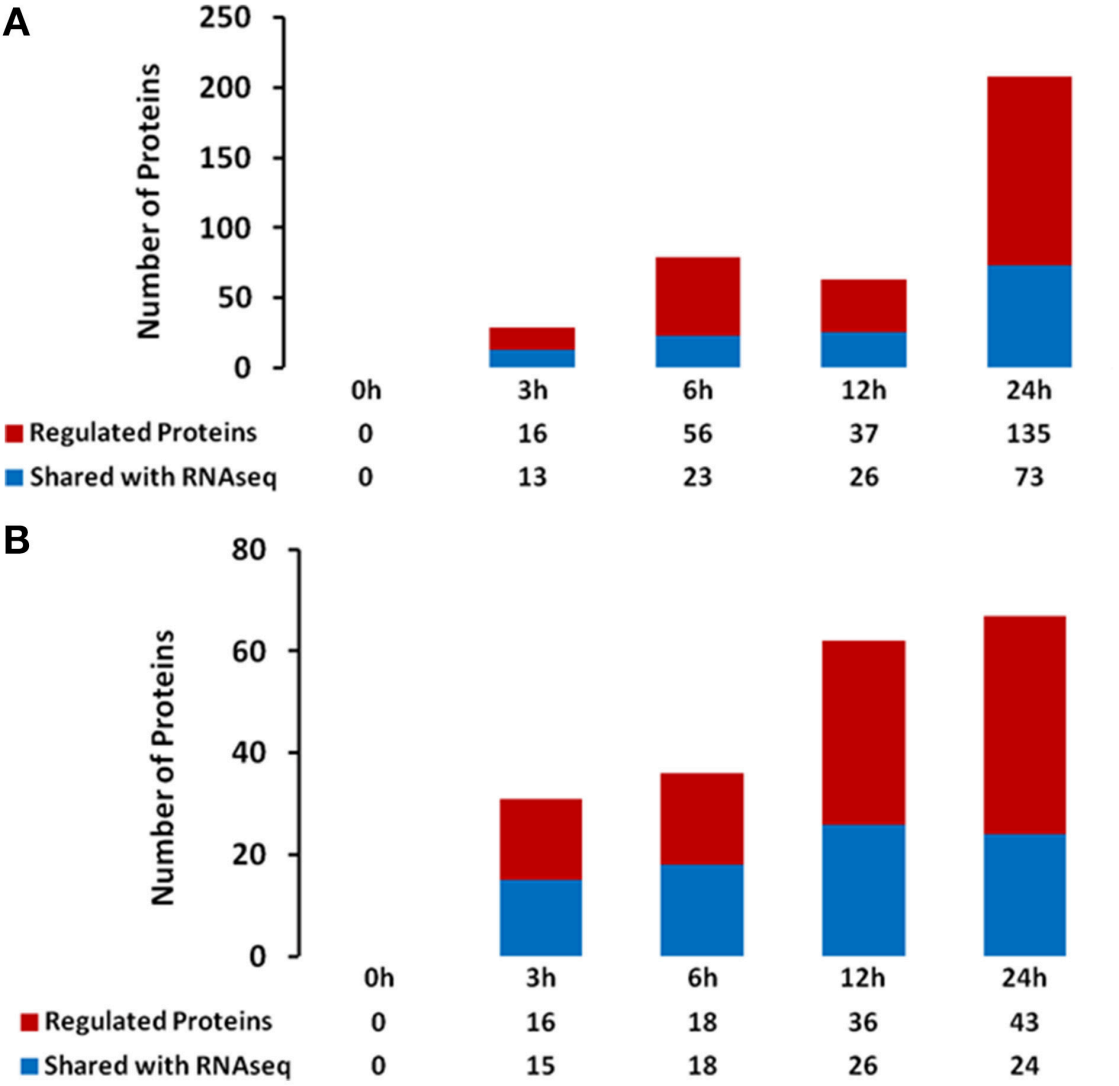
**Relationship between protein- and mRNA levels in heat-stressed soybean root hairs (A) and stripped roots (B)**.

In order to assess the relationship between the RNA and protein expression profiles, a pairwise comparison was made between the proteins and mRNA levels at the various treatments. This analysis gave a relatively low correlation value (0.2–0.79) between the mRNA and protein expression levels. However, on average, expression values for the mRNAs of 61 and 79% of the expressed proteins in RHs and STRs, respectively, were present in the transcriptome data set (Figure 6 and Table S2). Together, our proteomic data indicates that the majority of the expressed proteins have some, predicted role in coping with the heat stress, but also likely function to reprogram the transcriptional activity during heat stress conditions.

## Discussion

The predicted effects of continued climate change are complex but include effects on air and surface temperature, with coincident effects on water availability. In most regions, these effects are expected to significantly impact crop yields. Thus, it is important to understand the molecular mechanisms that allow plants to adapt and tolerate climate change induced stresses, including heat stress. Most of the studies to understand the plant responses to heat stress have focused on the leaf responses. For example, in different plant species both transcriptomic and proteomic analysis indicates that most of the molecular leaf responses are to protect the photosynthetic apparatus and to acquire general thermo-tolerance (Barah et al., 2013; Johnson et al., 2014; Liu et al., 2014; Sullivan et al., 2014). Despite the physiological relevance of roots, as well as the obvious effects that above ground processes have on root physiology, less attention has been placed on understanding how roots also respond to heat stress. Therefore, we undertook a detailed transcriptomic and proteomic study of the heat stress response in soybean roots. An important and unique aspect of our study was the examination of the effects on soybean root hairs, a single, differentiated, root epidermal cell.

Significant transcriptional and translational reprograming has been observed in different plant species grown under heat stress conditions (Zeller et al., 2009; Li et al., 2013; Johnson et al., 2014; Sullivan et al., 2014). Likewise, it was reported that this reprograming occurs very quickly, for instance after 10 min of treatment (Matsuura et al., 2010). Somewhat similar results were observed in our transcriptional analysis. Interestingly, we did observe that RHs showed a faster (e.g., 3 h) transcriptional and translational reprograming to heat stress than STRs. This observation suggests that studies of the response in single cells may reveal very different response kinetics and gene/protein expression responses than one can measure by studies of whole organs.

Over 2000 and 3000 genes were differentially regulated by heat in the RH and STR samples, respectively. A gene function enrichment analysis of these regulated genes suggest that heat has a strong effect on cellular metabolism, impacting genes involved in metabolic processes, response to environmental stimuli, transcriptional regulation, protein folding, chromatin remodeling, lipid, and ATP biosynthesis. It is important to note that these transcriptional responses are somewhat different from those reported for heat-stressed leaves, where the majority of the regulated genes are involved in thermo-tolerance and protection of the photosynthetic apparatus (Usadel et al., 2009; Barah et al., 2013; Zhu et al., 2013; Sullivan et al., 2014). The data indicate that there is a significant and early remodeling of the root transcriptional program in response to heat stress, presumably to maintain vital biological processes.

Under continuing predicted climate change conditions, it is important to develop stress-resistant crops. It was proposed that transcriptional network analysis can significantly contribute to the identification of new maker genes for potential use in plant breeding programs (Gehan et al., 2015). For example, previous regulatory network analysis in Arabidopsis and rice plants identified the TFs HSF, NF-X1, NF-Y, ZIM, bHLH, MYB, and DREBP as key to the transcriptional response to heat stress (Barah et al., 2013; Sarkar et al., 2014). Additionally, it was demonstrated that the rice transcriptional response to heat stress is mainly controlled by three gene regulatory modules (Sarkar et al., 2014). Similarly, our gene regulatory module analysis indicates that relatively few TFs are the main regulators of the heat stress response in soybean roots. However, these TFs are predicted to act in a combinatorial manner to control ten different regulatory modules. Specifically, these TFs are HSF (Glyma.03G157300), AP2/EREBP (Glyma.13G152000), MAD-box (Glyma.07G181600), and two WRKYs (Glyma.17G097900 and Glyma.19G020600). Interestingly, previous studies in other plant species support the participation of these TFs in the plant response to different abiotic stresses, including heat stress (Rushton et al., 2010; Lata and Prasad, 2011; Lenka et al., 2011). Further research focused on these specific TFs and the genes belonging to the identified modules that they control should provide additional mechanistic details to aid efforts to develop more heat tolerant soybean.

Although useful in predicted genes for further study, transcriptome analysis does not predict the level of expression of the encoded proteins. Hence, our proteomic analysis identified 357 proteins whose expression level was significantly affected by heat treatment. Interestingly, in contrast to the enrichment analysis of the transcriptome, the majority of proteins responding to heat are predicted to play a role in thermo-tolerance. For instance, we did identify heat-shock, class I and II, proteins, as well different peroxidases. Furthermore, it was reported that plants can modify membrane fluidity in response to heat stress (Hasanuzzaman et al., 2013). Consistent with this, we observed that different fatty acid desaturases were down-regulated. Other proteins which cause a significant downward expression of proteins in heat-stressed RHs include histones, which contribute to chromatin structure (Berger, 2007). Previous research also implicated histone modifications as playing an important role in plant adaptation to abiotic stresses (Pecinka and Scheid, 2012). For instance, it was reported that the occupancy of the histone H2A.Z, which tightly wraps the DNA, is reduced in heat-stressed Arabidopsis plants (Kumar and Wigge, 2010). This reduction in H2A.Z occupancy has a positive impact on the expression of heat-stress, induced genes (Kumar and Wigge, 2010). Thus, down-regulation of the soybean histone H2A by heat could contribute to an activation of chromatin regions supporting the expression of different genes in heat-stressed RHs.

Coupling transcriptomic and proteomic analysis of the same samples provides the opportunity to directly compare the data. As seen in a number of previous studies (Petrica et al., 2012; Stevens and Brown, 2013; Ponnala et al., 2014), there was a relatively low, overall correlation between the protein and mRNA expression levels. This is not unexpected since translation is governed by a variety of regulatory mechanisms, independent of transcription rate. These include the impacts of miRNA, mRNA half-life, translational rates, as well as protein turnover (Petrica et al., 2012).

Finally, signal dilution, which results from averaging the response from different cell types by sampling whole tissues, obscures the actual cellular response. Hence, it is impossible to discern the difference, for example, of a gene that is expressed at a low level in all cells from a gene that is expressed at very high level but only in few cells. Signal dilution can obscure key cellular responses to heat stress. Hence, sampling single cells is an excellent way to better define the cellular response to environmental changes (Hossain et al., 2015). In this study, we employed this approach with a specific focus on soybean root hairs, a single, differentiated cell type that plays a critical role in plant nutrition and water uptake. As expected, significant differences were noted when the response of RHs and STRs was compared. For instance, our gene/protein enrichment analysis indicated that after 6 h of treatment, most of the differentially regulated transcripts and proteins in RHs are directly involved in the root adaptation to the to heat stress. In contrast, the majority of the differentially regulated genes/proteins in STRs are involved in primary metabolism, with very few (~10) predicted to play a direct role in adaptation to heat stress. Our data reinforce the importance of single cell models to understand the molecular responses that allow soybean plants to adapt to heat stress. Likewise, our data also show that RHs are excellent model to discern the first root responses to environmental stimuli.

In conclusion, our results clearly demonstrate that roots respond strongly to heat stress and that the response of the single cell RHs is quite distinct from that of the remaining root tissue. The datasets generated provide a rich resource for further study and efforts to develop crop plants that can withstand the impacts of a changing climate.

## Author contributions

GS designed research. JB and NG generated the biological samples and performed experiments. NZ, TJ, and DX, performed the transcriptional analysis. KH, KW, JA, and LP, performed the proteomic analysis. CN and MI, performed data analysis, OVL, JB, and GS analyze the whole data set and wrote the paper.

## Funding

Research was funded by a grant from the Biological and Environmental Research Division, Department of Energy, Office of Science (Grant DE-SC0004898 to GS, DX, LP), as well as funding to GS from the United Soybean Board. A portion of this research was conducted under the Laboratory Directed Research and Development Program at PNNL, a multi-program national laboratory operated by Battelle for the U.S. Department of Energy under Contract DE-AC05-76RL01830. The work was performed at EMSL, a national scientific user facility sponsored by the Department of Energy's Office of Biological and Environmental Research and located at PNNL. OVL research is founded by a CONACyT (CONACyT# 219759 and 252260), PAPIIT-UNAM (PAPIIT# IA203815), and PAPCA-FES Iztacala (FESI-DIP-PAPCA-2014-3) grant.

### Conflict of interest statement

The authors declare that the research was conducted in the absence of any commercial or financial relationships that could be construed as a potential conflict of interest.

## Supplementary material

Figure S1 **Number of overlapping and non-overlapping heat-responsive genes among the different exposure time points in soybean root hairs**.

Figure S2 **qRT-PCR validation of heat stress-responsive genes**. A total of 15 randomly selected genes were used for qRT-PCR validation. Log2 fold change values (Treatment 40°C/Control 25°C) from the qRT-PCR data were plotted against Log2 (Treatment 40°C/Control 25°C) RNAseq values. Data are the average from two biological replicates.

Figure S3 **Gene regulatory modules identified in soybean heat-stressed root hairs**.

Figure S4 **MapMan classification of the regulated proteins in heat-stressed root hairs and stripped roots**.

Figure S5 **iTRAQ and LC-MS/MS experimental design**.

Table S1 **mRNA-seq analysis of 40 libraries generated from soybean control and heat-stressed root hairs and stripped roots**.

Table S2 **Correlation values for trancriptomic and proteomic expression levels measured in root hairs and stripped roots**.

Table S3 **Gene identification for each protein bellowing to the different overlapping and non-overlapping categories in RHs or STRs**.

Table S4 **Gene identification for each protein bellowing to the different overlapping and non-overlapping categories between RHs and STRs**.
