# Supplementary figures and images for: Soybean Roots Grown under Heat Stress Show Global Changes in Their Transcriptional and Proteomic Profiles
Source: Front Plant Sci. 2016 Apr 25;7:517. doi: 10.3389/fpls.2016.00517 (PMC4843095; doi:10.3389/fpls.2016.00517)

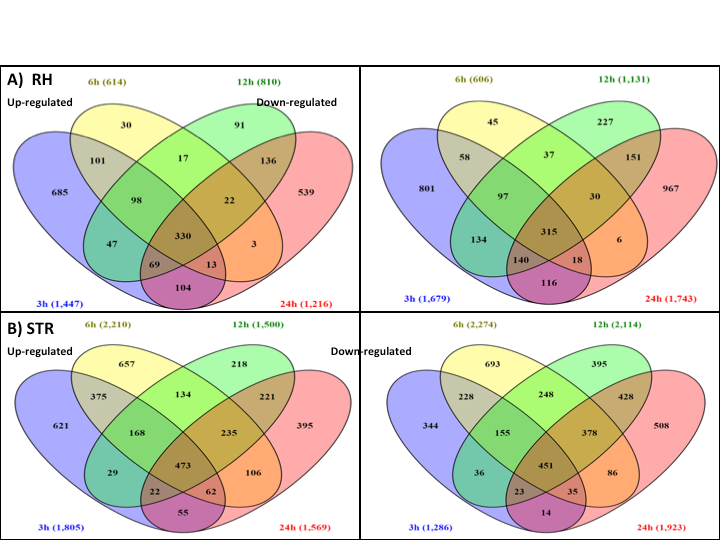

Supplement: Figure S1 — Number of overlapping and non-overlapping heat-responsive genes among the different exposure time points in soybean root hairs. [file Image1.TIF]

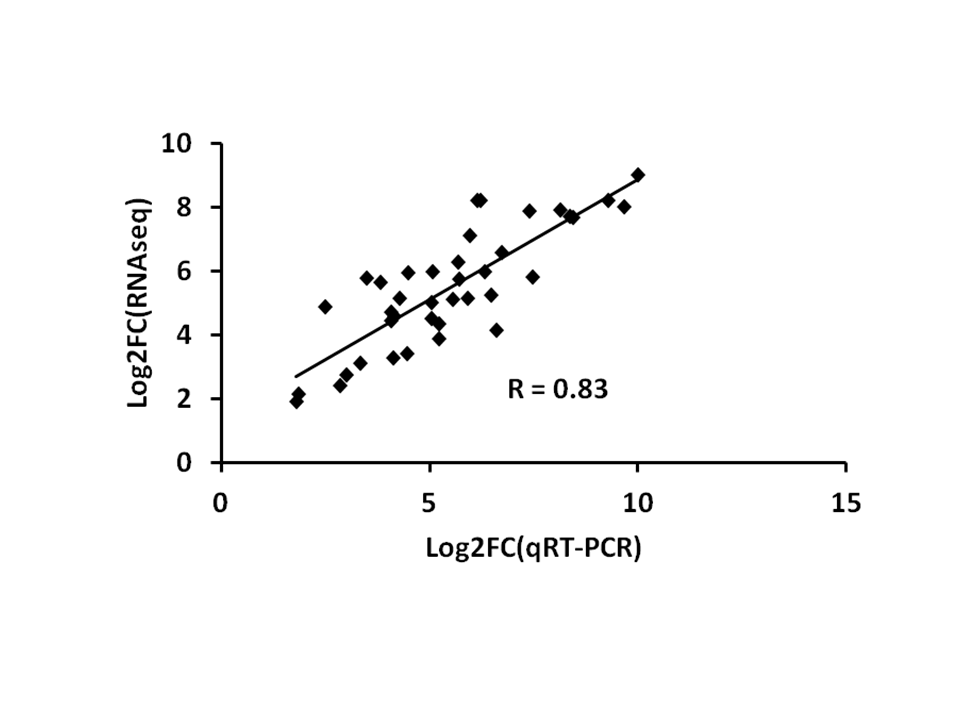

Supplement: Figure S2 — qRT-PCR validation of heat stress-responsive genes. A total of 15 randomly selected genes were used for qRT-PCR validation. Log2 fold change values (Treatment 40°C/Control 25°C) from the qRT-PCR data were plotted against Log2 (Treatment 40°C/Control 25°C) RNAseq values. Data are the average from two biological replicates. [file Image2.TIF]

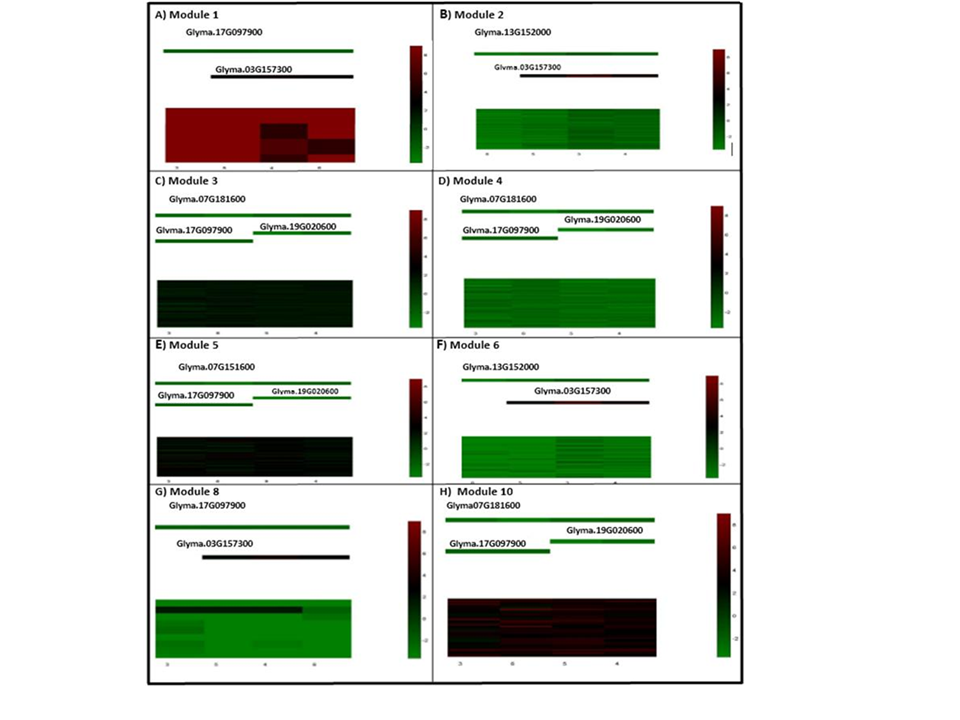

Supplement: Figure S3 — Gene regulatory modules identified in soybean heat-stressed root hairs. [file Image3.TIF]

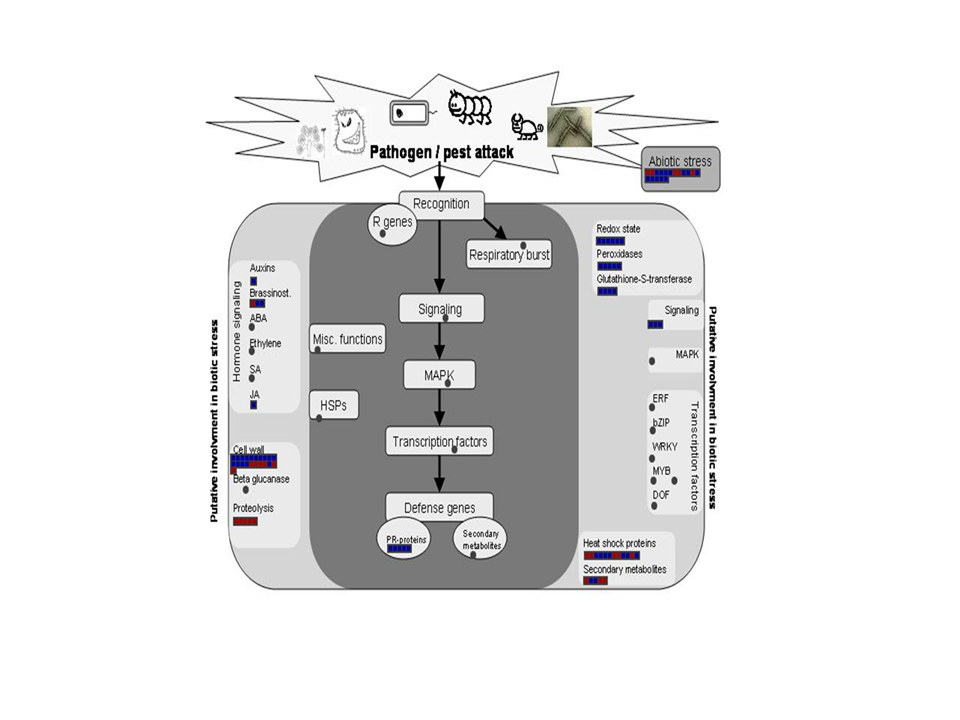

Supplement: Figure S4 — MapMan classification of the regulated proteins in heat-stressed root hairs and stripped roots. [file Image4.tif]

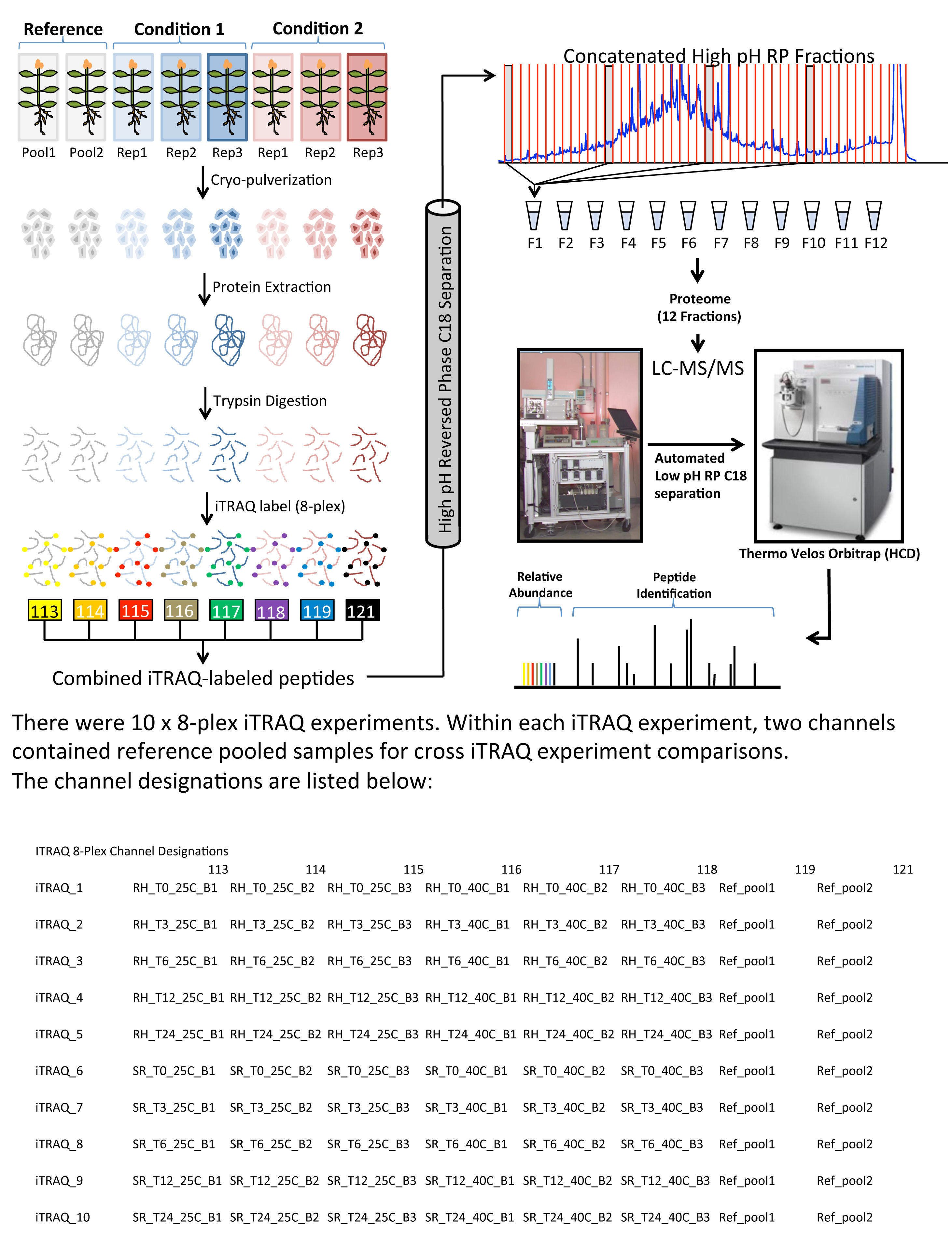

Supplement: Figure S5 — iTRAQ and LC-MS/MS experimental design. [file Image5.jpg]
