# Supplementary material for: Soybean Roots Grown under Heat Stress Show Global Changes in Their Transcriptional and Proteomic Profiles
Source: Front Plant Sci. 2016 Apr 25;7:517. doi: 10.3389/fpls.2016.00517 (PMC4843095; doi:10.3389/fpls.2016.00517)
Supplement: Table S1 — mRNA-seq analysis of 40 libraries generated from soybean control and heat-stressed root hairs and stripped roots. [file Table1.DOCX]

**Supplementary Table 1:** mRNA-seq analysis of 40 libraries generated from soybean control and heat-stressed root hairs and stripped roots.

| Cell type | Raw reads^a^ | Good quality reads^a^ | Mapped reads^a^ | Uniquely mapped reads^a^ | Multi-mapping reads^a,b^ |
| --- | --- | --- | --- | --- | --- |
| Root Hairs | 673,819,026 | 585,811,470 | 509,022,644 | 482,471,224 | 26,551,420 |
| Stripped Root | 754,723,285 | 613,110,894 | 544,509,934 | 515,452,180 | 29,057,754 |

^a^ Values represent the total reads from 20 libraries of each cell type.

^b^ Reads that map in at least ten different genomic locations.
