# Supplementary material for: Soybean Roots Grown under Heat Stress Show Global Changes in Their Transcriptional and Proteomic Profiles
Source: Front Plant Sci. 2016 Apr 25;7:517. doi: 10.3389/fpls.2016.00517 (PMC4843095; doi:10.3389/fpls.2016.00517)
Supplement: Table S2 — Correlation values for trancriptomic and proteomic expression levels measured in root hairs and stripped roots. [file Table2.DOCX]

**Table S2:** Correlation values for root hairs and stripped roots trancriptomic and proteomic expression levels. (A) Correlation values for each time point and tissue. (B) Correlation values for each gene expressed at all time points. RH: 8; STR: 10.

**(A)**

| **Time points and RH tissue** | **Correlation values** |
| --- | --- |
| 3H | 0.005 |
| 6H | 0.594 |
| 12H | 0.639 |
| 24H | 0.798 |
| **Time points and STR tissue** | **Correlation values** |
| 3H | -0.571 |
| 6H | 0.040 |
| 12H | -0.192 |
| 24H | 0.427 |

**(B)**

| **RH all time point, 8 genes** | **Correlation Values** |
| --- | --- |
| Glyma04g05720 | -0.860 |
| Glyma06g05740 | 0.014 |
| Glyma07g32050 | -0.598 |
| Glyma13g24440 | -0.536 |
| Glyma13g24480 | -0.562 |
| Glyma13g24490 | -0.082 |
| Glyma18g43430 | -0.929 |
| Glyma20g01930 | -0.179 |
| **STR all time point, 10 genes** | **Correlation Values** |
| Glyma04g05720 | -0.61 |
| Glyma06g05740 | -0.26 |
| Glyma07g32050 | -0.45 |
| Glyma08g07330 | -0.03 |
| Glyma08g07340 | 0.08 |
| Glyma08g07350 | -0.11 |
| Glyma08g22630 | -0.09 |
| Glyma12g01580 | 0.76 |
| Glyma13g24490 | -0.89 |
| Glyma20g01930 | 0.60 |
